# Examining the Influence of Integrated Home and Community Care Programs on Quadruple Aim and Health Equity Outcomes Across the Health Care System: A Scoping Review

**DOI:** 10.5334/ijic.9896

**Published:** 2026-03-19

**Authors:** Margaret E. Saari, Ryan McLeod, Marie Lauro, Paul Holyoke, Justine L. Giosa

**Affiliations:** 1 SE Research Centre, SE Health, Markham, Ontario, Canada; 2 Lawrence Bloomberg Faculty of Nursing, University of Toronto, Toronto, Ontario, Canada; 3 School of Public Health Sciences, University of Waterloo, Waterloo, Ontario, Canada

**Keywords:** integrated care, home care, community care, health services research, scoping review, quality of health care

## Abstract

**Introduction:**

Integrated approaches have been promoted as a solution to challenges in delivering high-quality care for aging populations. Despite their theorized benefits, the system-wide influence of integrated home and community care models remains poorly understood.

**Methods:**

We systematically searched MEDLINE (Ovid), CINAHL (EBSCO) and AgeLine (EBSCO) for studies examining the influence of integrated home and community care programs on outcomes aligned with the Quadruple Aim and Health Equity framework.

**Results:**

Forty-seven peer-reviewed articles were included, representing five program types: comprehensive coordinated care (n = 6), integrated palliative care (n = 23), preventative care (n = 4), restorative care (n = 3), and transitional care (n = 11). Most studies focused on value outcomes, particularly in hospital (n = 36) and emergency services (n = 22). Patient and caregiver experience was assessed in 11 studies, while provider experience (n = 1), health equity (n = 2) and population health (n = 0) were rarely examined. Eleven studies evaluated system-level influence; 23 focused on community-dwelling care recipients.

**Discussion:**

The emphasis on cost-related outcomes reflects prevailing austerity and value-based rhetoric, which may shape research funding, priorities and attention.

**Conclusion:**

Future research should adopt a balanced approach, incorporating all Quadruple Aim and Health Equity domains. A learning health system model, linking data, knowledge and practice, is recommended to enable continuous improvement and resource alignment.

## Introduction

The capacity of Canada’s health system to provide high-quality complex care to an aging population is under pressure, owing in part to the increased prevalence of individuals with complex social needs as well as multiple and chronic health conditions [1,2,3]. Most existing care models are fragmented, lacking processes and communication channels necessary to facilitate high-quality care [4] to respond to needs. In response to shifts in patterns of needs, publicly funded health care systems worldwide are in search of transformative solutions to improve population health and the delivery of long-term care services, while ensuring that limited resources and services are used effectively [5]. Novel care models that support both client autonomy, and the overwhelming preference of Canadians to age in their homes and communities, are needed to meet demand [6,7,8,9,10]. Shifting towards an integrated approach to care delivery has been suggested as an important first step in health system reform, fostering more comprehensive and coordinated care across health and social services, that responds holistically to intersecting needs of older adults [11,12,13].

In Canada, calls for increased investment in integrated approaches to home and community care date back to the Romanow Commission [14], with attention heightened since the onset of the COVID-19 pandemic [15,16]. Home and community care includes delivery of both home health care and supportive care services intended to facilitate clients’ ability to age well in their communities [17]. Home health care includes care and services provided by regulated health professionals, along with the medical equipment and supplies required to provide care. Supportive care services focus on supporting aspects of daily living integral to remaining in the community, including completion of personal care, housekeeping, and meal preparation, as well as transportation, social activities and community engagement. Despite promotion of integrated home and community care programs as a solution to aging-related health system challenges [13] and additional funding commitments and policy attention [18,19], the influence of these programs on the broader health system is not well documented or understood.

System-level evidence related to the impact of integrated home and community care programs is limited. Previous research examining the intersectoral impacts of integrated home and community care has focused on program impacts on facility-based care such as hospitals and nursing homes [20]. No reviews could be located that examined effects of integrated home and community care programs across the broader health care system, including primary care and unpaid caregivers. Existing evidence syntheses also do not reflect recent changes to the community-based care environment, such as increasing complexity of client needs [1], growth of telemedicine and digital health [21], increasing ethnocultural diversity of older adults [22,23,24], or expansion of research on integrated health and social care [25]. Reviews that have taken a broader lens to examine effects of other home-based services on the health system have focused mostly on individual-level clinical outcomes and cost-effectiveness [26,27]; providing only a partial measurement of the quality and impact of healthcare services. Health system planning, including program and policy development, implementation, delivery and evaluation, requires a thorough understanding of the reach, acceptability, effectiveness, efficiency and sustainability of integrated home and community care programs to ensure the system can meet the needs of aging Canadians, and those providing essential care and support [28,29].

The Quadruple Aim is an internationally recognized healthcare improvement framework that delineates four focus areas for health system redesign and performance improvement: population health, value, provider experience, and patient experience [30,31]. In Canada, the Institute of Health Services and Policy Research has proposed the use of the *Quadruple Aim and Health Equity* as a guide for health system transformation [32]. In their most recent strategic plan, health equity has been intentionally positioned as crosscutting the other four domains, emphasizing the importance of ensuring health system improvement efforts do not exacerbate existing health disparities [32,33]. Along with patient experience, we include the experience of caregivers given the integral role they play in addressing older adults’ needs and the identified health systems navigation challenges older adults face [34]. This framework offers a clear structure for monitoring and evaluating novel programs and reforms that promote both high-quality care and Canadians’ autonomy to live and age independently, in their setting of choice.

The objective of this evidence synthesis was to map the nature and extent of the extant literature on the influence of integrated home and community care programs across the health system, through the lens of the Quadruple Aim and Health Equity framework. Synthesizing the literature in this way is intended to provide a critical evidentiary base for supporting the development, spread and scale of age-friendly solutions to current health system challenges and identify the gaps and opportunities for future research and evaluation.

## Methods

In light of growing national and international focus on the design and delivery of integrated care [35,36], and the need to identify pertinent gaps to guide future research [37], a scoping review was selected to synthesize evidence on this topic. Following Arksey & O’Malley’s six-step methodology [38] with Levac et al.’s [39] refinements, we engaged point-of-care clinicians, home care leaders and health system partners to establish the research question, review preliminary results, formulate and refine messaging and explore implications for future research and practice. This review is reported in alignment with the Preferred Reporting Items for Systematic Reviews and Meta-Analyses Extension for Scoping Reviews (PRISMA-ScR) guideline [40]. As recommended by the Joanna Briggs Institute, critical appraisal of included studies was not conducted [37].

### Protocol and Registration

This review was conducted in accordance with an *a priori* protocol registered on the Open Science Framework on June 23, 2023 (https://doi.org/10.17605/OSF.IO/YVK5S).

#### Stage 1: Identifying the research question

The need for this scoping review was identified during the final phases of a larger research study focused on developing a model of integrated home and community care to address the holistic care needs of older adults and support aging-in-place [1,15]. Plans to assess feasibility of the newly developed care model through economic modelling were deferred due to challenges identifying relevant and consolidated literature examining the influence of integrated home and community care to develop and validate model cost assumptions. To explore the supposition that providing integrated care leads to improved client and system-level outcomes, in partnership with study collaborators, the following overarching research question was developed to guide this review, **‘*What influence do integrated home and community care programs have on Quadruple Aim and Health Equity outcomes across the health system?*’**. Specifically, we aimed to better understand the influence of integrated home and community care programs on 1) patient and caregiver experience, 2) population health, 3) value (i.e., costs of care), 4) provider experience and 5) health equity, as well as the types of evidence available. We also wanted to better understand research and evaluation priorities in this space by analyzing the volume of evidence associated with each of the Quadruple Aim and Health Equity domains.

#### Stage 2: Identifying relevant studies

A comprehensive search strategy (Supplementary File 1) was developed with guidance from a public health librarian. The search strategy was executed on July 10th, 2023, and run across three major databases in the fields of biomedicine, nursing and allied health, and gerontology: MEDLINE (Ovid), CINAHL (EBSCO) and AgeLine (EBSCO). The search was conducted using keywords designed to capture the following inclusion criteria:

**Participants**: Adults aged 18 years and older who were recipients of formal home care services integrated with community-based services including primary care, community pharmacy, community support services, or community mental health services. Formal home care services were operationally defined as the provision of one or more of the following in-home services: nursing, personal care, physiotherapy, occupational therapy, speech therapy, social work, dietetic services, respite services, and homemaking.

**Context**: Studies examining outcomes of integrated home and community care programs across five health system settings, including:

- Hospital-based care: Care and services provided during hospital admission, including care provided after acute admission is complete (e.g., alternate level of care, delayed discharge).
- Emergency medical services: Urgent care and services provided to stabilize and pre-treat illness or injury, including emergency department visits, paramedic and ambulance service use, and emergency dispatch.
- Primary care: Routine and comprehensive care and services provided in a primary care setting including care delivered by family physicians, nurse practitioners, and interdisciplinary teams.
- Informal care: Care provided by unpaid caregivers such as the client’s family and/or social network.
- Facility-based long-term care: Nursing, personal care and other services provided in a facility such as long-term care homes, assisted living, retirement homes, skilled nursing facilities, complex continuing care.

**Concept**: Outcome measures aligned with the Quadruple Aim and Health Equity domains including value, patient and caregiver experience, population health, care team (provider) experience, and health equity.

**Types of sources**: Peer-reviewed primary sources including quantitative, qualitative, and mixed methods designs, as well as evidence synthesis articles including scoping, systematic, rapid, umbrella, narrative, integrative, or realist reviews.

**Publication language**: No limits were placed on language during title and abstract review to understand linguistic variation in this topic. However, only English-language full-text articles were included to allow all authors to participate fully in the review.

**Timeframe**: Sources from 2013 onwards were included to reflect the substantial growth in research focused on integrated health and social care since then [25,41].

To ensure comprehensiveness of the search strategy, manual searches of reference lists of included articles, as well as prominent integrated care journals, including: the International Journal of Integrated Care, the Journal of Integrated Care, Health and Social Care in the Community, and the International Journal of Care Coordination were conducted.

#### Stage 3: Selecting studies

A two-stage screening process was employed to select studies. Covidence was used to manage the screening process and facilitate duplicate removal. Thirty-one duplicates were removed manually. Two reviewers conducted independent screens of 50 articles to pilot test inclusion and exclusion criteria, to ensure consistency in screening and robustness of eligibility criteria. Titles and abstracts of all articles identified through the search were screened for relevance by one of three reviewers (MS, ML, RM). Articles published in 2013–2023 in English that assessed the influence of a program that integrated formal home care with community-based services, and explored outcomes related to the Quadruple Aim and Health Equity framework in any of the five health services settings of interest were included.

Full text copies of relevant articles were retrieved, imported into Covidence, and independently reviewed by two reviewers (ML, RM). Articles meeting any of the following criteria were excluded:

- examined a pediatric population (<18 years);
- program did not include home and community care, focused on home-based visits but was independent of home care, or did not include integration with community-based services;
- not a peer-reviewed study; or
- did not assess an outcome related to value, patient and caregiver experience, population health, care team (provider) experience or health equity.

Two reviewers (MS, ML) piloted the inclusion/exclusion criteria and data extraction template, independently reviewing a small sample of articles, then meeting to discuss and resolve conflicts to ensure consistency between reviewers. The review team continued to meet regularly to discuss challenges, review conflicts, and ensure all relevant literature was included. The team decided that articles that could not be conclusively identified as integrated home and community care due to a lack of clear and detailed descriptions of the integrated care program under study would be excluded.

#### Stage 4: Charting the data

Data from included studies were extracted by three research team members (RM, ML, MS). For each article, two authors independently extracted data. The team met regularly to discuss conflicts and reach consensus. Data were extracted using a standardized instrument (Supplementary File 2).

#### Stage 5: Collating, summarizing, and reporting the results

**Figure 1:**
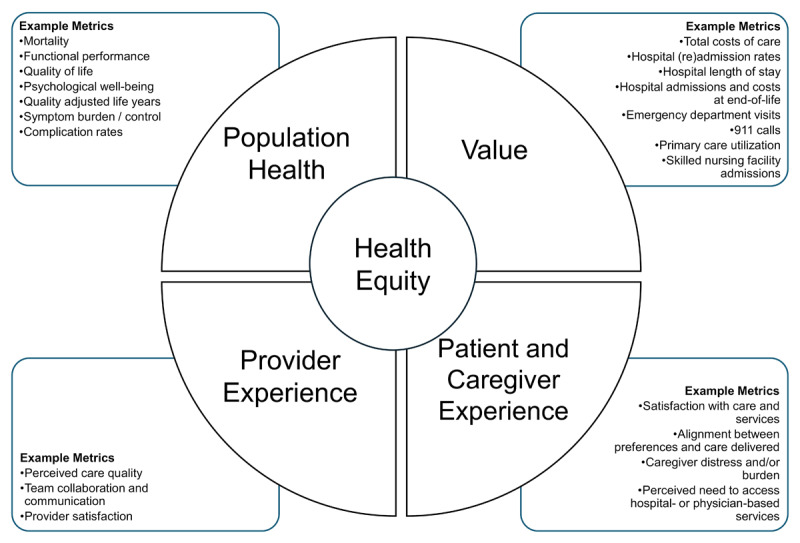
Metrics mapped to the Quadruple Aim and Health Equity domains.

Extracted data were tabled to examine the extent, range, and nature of the evidence on the influence of integrated home and community care programs on the health system. Analysis of extracted data occurred on three levels: 1) program details were used to categorize identified programs, 2) outcome metrics were examined and summarized to assess whether program impacts were positive, negative, or mixed within each setting and 3) metrics were mapped to the Quadruple Aim and Health Equity domains (see Figure 1) to assess the volume of evidence available for each domain. A narrative summary was generated to further describe findings according to the research questions [40].

#### Stage 6: Consultation

Three virtual consultation sessions were conducted between April and August 2024. The first session engaged cross-functional leaders representing finance, strategy, legal, privacy and compliance, operations, and policy perspectives from a large Canadian home care organization with experience in delivering integrated home and community care. Two subsequent sessions involved leaders from Ontario’s newly established integrated care organizations.

Potential participants received email invitations from the lead researcher outlining the session agenda, providing an opportunity to seek clarification regarding study participation, and requesting selection of a preferred date. A pre-read package was distributed in advance, which included an overview of the Quadruple Aim and Health Equity framework, key study definitions, and a list of identified programs. At the outset of each session, participants were informed that discussions would be recorded and transcribed for research purposes and were given the option to decline participation. Each consultation began with a presentation of preliminary findings, followed by structured discussions addressing: 1) alignment of findings with their professional experience, 2) the types of information requested by funders when proposing new integrated care initiatives and 3) the evidence necessary to support uptake, scale and spread of integrated home and community care programs.

Transcripts were analyzed using qualitative content analysis [42] to identify themes and conclusions that reinforced, challenged, or refined preliminary scoping review findings. This process ensured that interpretation was grounded in practical, context-specific lived experience.

#### Ethical Considerations

Ethical review was sought from the Southlake Health Research Ethics Board (S-034-2324) and deemed exempt under Article 2.5 of the Tri-Council Policy Statement: Ethical Conduct for Research Involving Humans (TCPS 2). The study was conducted in accordance with TCPS 2 ethical standards.

## Findings

### Search results

**Figure 2:**
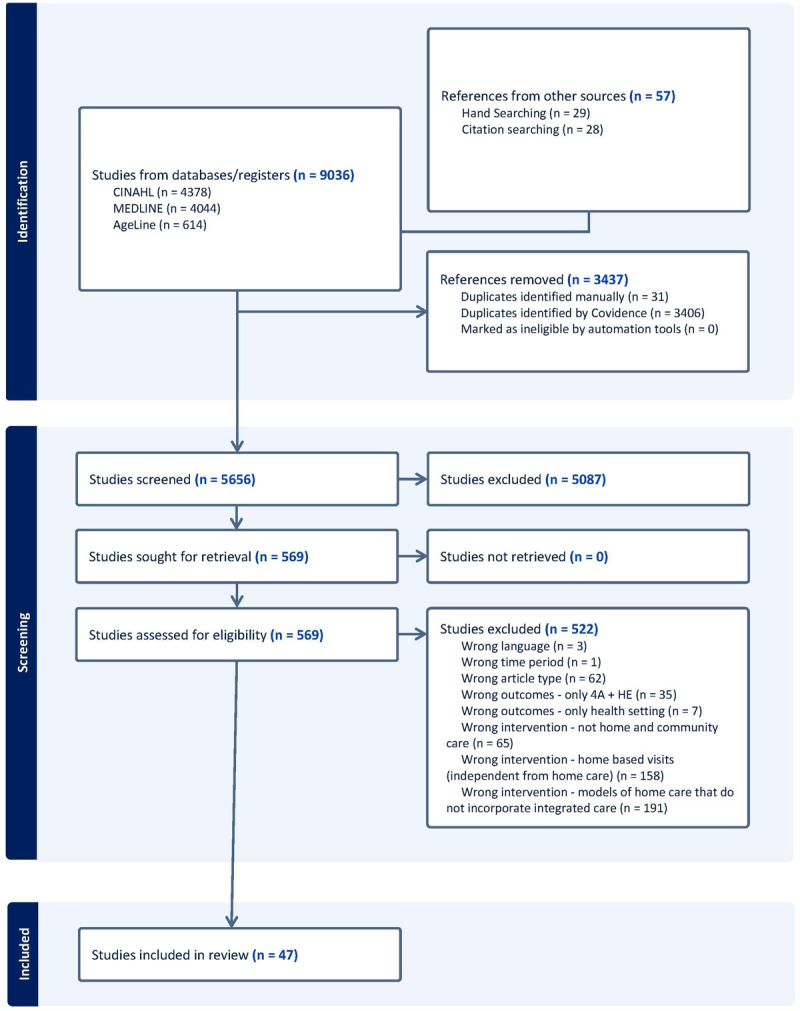
PRISMA flow diagram.

Database searches yielded 9,036 records, with 5,656 unique titles and abstracts screened after removing duplicates. Of these, 569 met criteria for full-text review, resulting in 47 peer-reviewed articles included in the final analysis. See Figure 2 for the PRIMSA flowchart of study selection processes.

### Publication characteristics

Characteristics of included studies are presented in Supplementary File 3. Included studies employed qualitative (n = 3), quantitative (n = 42), and mixed methods (n = 2) study designs. Articles were published across 29 journals representing biomedicine, nursing and allied health, and gerontology. Included studies were conducted across 16 countries, with nearly half of studies being conducted in North America including the United States (n = 15) and Canada (n = 7), with the remaining studies (n = 25) based in Singapore, Italy, Sweden, Australia, China, Denmark, France, Greece, Israel, Japan, the Netherlands, Switzerland, Spain, Taiwan, and the United Kingdom.

### Program types and description

Details regarding program care team composition and activities were extracted from each article (Supplementary File 4), focusing on the team structure, services provided, and any additional program structures that supported integration. Using this information, articles were categorized into five unique program types: 1) **comprehensive coordinated care** (n = 6) programs which coordinated medical, social and long-term care services for older adults with complex needs receiving care in their homes and communities; 2) **integrated palliative care** (n = 23) programs which brought together home-based care with specialized palliative care services; 3) **preventative care** (n = 4) programs which integrated home-based care with preventative care practices including primary care, 4) **restorative care** (n = 3) programs which provided home based-care focused on rehabilitation and restoring functional ability; and 5) **transitional care** (n = 11) programs which provided home-based care to individuals during hospital-to-home transitions.

### Influence of integrated home and community care on the overall health system

Across included studies, 11 articles examined the influence of integrated home and community care on the overall health system, reporting generally positive findings. Most identified reduced total care costs [43,44,45,46,47,48,49,50,51], or no net cost-reduction [52]. However, Yu and colleagues [53] suggest that overall costs are not reduced but rather redistributed. Improvement in health system navigation (i.e., access to services) was also reported [43].

Twenty-three articles examined the influence of these programs on community-dwelling care recipients, reporting benefits including improved satisfaction with care [54,55], improved therapeutic relationships with care providers [44,56], respect for preferences for setting of care [53,57] and increased completion of advanced care directives [58,59]. Integrated care programs also demonstrated varying degrees of proficiency in improving population health outcomes including quality of life [43,44,45,48,60,61,62], mortality [47,59,63,64,65,66], functional performance [60,67,68,69], and symptomology [54,55,61,62,68,70].

### Quadruple Aim and Health Equity outcomes

**Figure 3:**
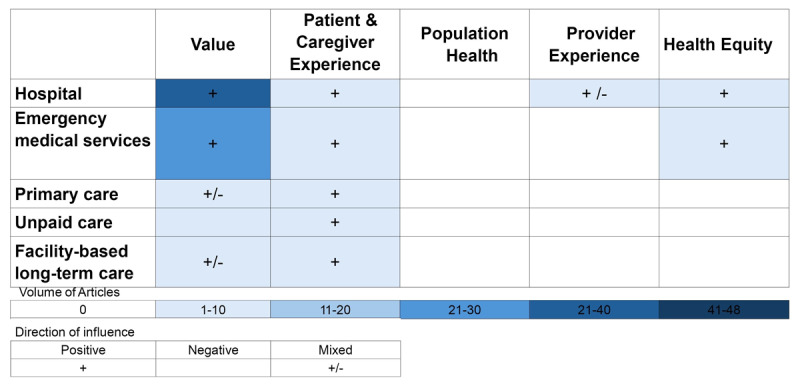
Number of articles investigating Quadruple Aim & Health Equity related outcomes by health setting.

Figure 3 summarizes the volume of evidence available for each Quadruple Aim and Health Equity domain, as well as the direction of influence in each care setting. Many articles examined the influence of programs on more than one setting. Among the studies included, 75% (n = 36) explored value outcomes in hospital settings, and 48% (n = 22) did so in emergency medical services (EMS). Outcomes related to patient and caregiver experience were investigated across all care settings (hospital, n = 3; EMS, n = 1; primary care, n = 1; unpaid care, n = 5; facility-based long-term care, n = 1). Health equity outcomes were examined by a single article in hospital (n = 1) and EMS (n = 1) settings, while the influence of integrated home and community care on population health outcomes in the settings of interest were not examined. The following section provides details on metrics used and direction of influence within each of the Quadruple Aim and Health Equity domains.

### Value

Thirty-six articles examined the influence of integrated home and community care programs on hospital service utilization, employing a variety of metrics and reporting mostly positive outcomes. Integrated programs were associated with reductions in hospital admissions [43,44,46,48,49,50,54,55,58,59,66,71,72,73,74,75], re-admissions [55,70,76,77,78,79], length of stay [44,46,49,50,53,59,61,64,67,71,74,79,80,81,82], and overall hospital resource use [44,45,77]. Several studies also noted lower hospital mortality rates [52,58,71,83]. However, findings were not consistent; some studies found no significant differences in admissions [57], readmissions [63,69], mean number of hospitalizations [47], or hospital days [69].

Emergency care service utilization was assessed in twenty-two studies. Reported outcomes included reductions in emergency department (ED) visit rates [71,84], overall ED visits [85,86] likelihood of ED attendance [50,73,77,87], average number of ED visits [54], ED use in the final months of life [51,87], and reliance on emergency transportation or 911 services [43,45]. However, some studies found no significant differences in ED visit rates [59] or number of visits [46,79], and one study reported increased odds of 30-day observation or ED return [76].

Primary care utilization was examined in three articles. Brännström & Boman [61] reported reductions in physician and nursing visits, while two studies found increased primary care use [45,71]. Only one article examined informal care [53], noting that caregivers of palliative patients who died at home provided twice as many unpaid hours compared to those caring for patients who died in hospital. Finally, one study evaluated facility-based long-term care, with no difference in admission rates observed [66].

### Patient & caregiver experience

Patient and caregiver experience was examined across eleven articles. Compton and colleagues [56] reported participants experienced reduced reliance on hospital, ED, or primary care services and expressed confidence in their ability to age in place. Two studies explored the influence of integrated home and community care location, finding community-based integrated programs improved alignment between patient and family preferences and actual care delivery, for example, higher rates of home deaths among patients preferring to die at home [66] and fewer avoidable hospitalizations [44].

Five articles assessed caregiver experience among those providing unpaid care, reporting perceived reductions in caregiver strain and distress [60], burden [62,68], stressors related to routine care demands [88], and depressive symptoms [68]. However, one study found that caregivers of persons receiving integrated palliative cancer care were less likely to feel safe providing care at home compared to those in facility-based palliative care [57].

### Population health

No articles were identified that compared population health outcomes of integrated home and community care programs against outcomes in the specific settings of interest.

### Provider experience

Only one article examined the influence of integrated home and community care on provider experience in health settings of interest, reporting that the program improved collaboration between hospital and community stroke services, despite persistent data integration and information gaps [80].

### Health equity

Similarly, only one article assessed the influence of integrated care programs on outcomes for equity-deserving groups. Kramer et al. [73] reported comparable reductions in hospital admissions and ED visits for rural Indian Health Service (IHS) beneficiaries and non-IHS beneficiaries receiving integrated home-based palliative care in the United States.

### Consultations

A total of 67 health leaders participated in three consultation sessions (Session 1: n = 19; Session 2: n = 24; Session 3: n = 24). Participants identified barriers to comprehensive research and evaluation on the system-wide influence of integrated home and community care programs, emphasizing limited resources and capacity for evidence-informed decision-making, and highlighted opportunities to build learning communities. Rigid funding requirements favoring cost-reduction were noted as a possible explanation for the skew in available literature. Participants also emphasized the need to scale and spread evidence from this review to inform decisions and investment in scientific and technological infrastructure, aiming to strengthen understanding of integrated care impacts—particularly provider experience, including staffing, cost-sharing, financial sustainability, and other key enablers.

## Discussion

Through this scoping review, we synthesized 47 peer-reviewed articles using diverse evaluation methods and metrics to better understand the influence of integrated home and community care programs on health systems. Applying the Quadruple Aim and Health Equity lens revealed an evidentiary focus on value (cost and cost-reduction) outcomes, with notable gaps in other domains of healthcare quality including population health, patient and caregiver experience and provider experience. Most studies examined the influence of these programs on acute care, with limited attention to primary care, unpaid caregivers, or facility-based long-term care outcomes. This review contributes to the growing evidence base aimed at informing decision-makers and advancing health system transformation to better align care with people’s preferences.

Value outcomes dominated the literature, particularly in relation to hospitals (n = 36) and emergency services (n = 22), validating health system leaders’ observations that cost-containment narratives shape evaluation priorities [89]. Most articles demonstrated reductions in hospital costs [43,45,48,71], utilization of hospital and emergency services [50,71,73,77,84] and hospital deaths [45,52,54,58,71,83]. Importantly, Yu et al. [53] highlighted the complexity of the matter, suggesting that costs are not reduced, but rather redistributed to patients and unpaid caregivers, who bear additional financial and time burdens.

Beyond system-level benefits, some studies demonstrated improvements in patient and caregiver experience, including care delivered in preferred settings [44,57,62] and reduced caregiver distress [60,88], burden [62,68], and depressive symptoms [68]. These effects were less pronounced in complex care situations such as palliative care [57]. Provider experience and health equity outcomes were scarcely evaluated, though Markle-Reid et al. [43] reported strong collaborations among patients, providers and community services improved quality of care, and providers identified data integration as a key enable for team communication.

Although integrated care has been widely promoted as a solution to aging-related health and care challenges, current evidence does not conclusively demonstrate high-quality care or optimal system performance. Findings often conflate cost reductions achieved in hospitals with genuine, system-wide improvement [11,12,13]. More comprehensive research is needed on the system-wide influence of integrated home and community care programs, particularly their influence on provider experiences and health equity. Consultations with health leaders suggest that the published literature reflects the funder reporting requirements, shaped by prevalent austerity and value-based rhetoric, and concerns of financial sustainability [90,91,92,93]. This shifting prioritization towards generating “value” seemingly mirrors the shifting predominance of research focus from an early focus on palliative care towards transitional programs developed, in part, to shift care, and therefore cost, out of hospitals.

Future efforts should adopt a more balanced approach by incorporating all domains of the Quadruple Aim alongside health equity considerations. This will enable a more holistic understanding of program impacts at both system and organizational levels. Health system leaders can apply these findings by using the Quadruple Aim and Health Equity framework as a decision-support tool for balanced priority-setting and strategic planning. There is also an opportunity for health organizations to partner meaningfully with researchers to advance thought leadership in this area. Such partnerships can strengthen decision-making by generating high-quality evidence that demonstrates the influence of integrated programs across sectors and care settings. Achieving this will require engagement with system users and collaboration between care teams and applied health services researchers who possess the skills and competencies to deliver high-quality evaluations, facilitate rapid learning cycles and mobilize knowledge for scale and spread [94,95,96,97]. These relationships can be fostered through learning health systems approaches, which create intentional opportunities for collaborative learning and knowledge development to generate practical, implementable solutions [98,99,100]. Approaches such as learning communities are particularly suited to addressing these gaps because they enable iterative feedback, shared priority-setting, and evidence-informed decision-making.

Through our consultations, health system leaders highlighted the challenges of meeting funder reporting requirements while expressing a desire for more standardized and holistic data collection and synthesis support. Robust research, evaluation, and quality improvement infrastructure will be essential to design, implement, and mobilize high-quality outcome evaluations for evidence-informed planning, decision-making, and implementation of integrated home and community care programs. As part of this infrastructure, the creation of inclusive ‘learning communities’ – bringing together care providers, patients, caregivers, applied health researchers, and decision-makers – can facilitate development of shared integrated care metrics, clarify and negotiate priorities among collaborators with diverse expertise, and foster trust [100].

### Strengths and limitations

This scoping review has several strengths, including a comprehensive and systematic search strategy, application of established methodological frameworks, and inclusion of diverse study designs to map the breadth of evidence on integrated home and community care. Engagement of health system leaders to support knowledge mobilization, combined with framing findings through the lens of the internationally recognized Quadruple Aim and Health Equity framework, enhances the relevance and applicability of findings by diverse audiences.

However, certain limitations should be noted. The exclusion of non-English language publications and grey literature may have resulted in missed insights. Considerable variation in definitions of integrated home and community care, and descriptions of program characteristics may have influenced the scope and comparability of available evidence. Additionally, this review did not include direct involvement of patients or family caregivers in shaping the research question, analysis, or interpretation. While consultations engaged health system leaders and providers with operational experience, the absence of perspectives from those receiving care represents an important gap. Future research should prioritize meaningful engagement of people with lived experience to ensure findings reflect their needs and priorities. Finally, the lack of quality appraisal, although typical for scoping reviews, limits our interpretation of program influence to directional trends rather than strength of effect.

## Conclusion

This scoping review calls attention to the current prioritization of economic considerations in health services research and healthcare policy and decision-making. Although the published literature does not permit conclusive and unequivocal promotion of integrated approaches to home and community care as an age-friendly solution to current challenges, the evidence highlights the potential for cost-reductions and improved care experiences for patients and caregivers. To address the practice-knowledge gap in Quadruple Aim and Health Equity outcomes, a learning health systems approach is recommended that aligns the necessary infrastructure, resources, perspectives and expertise to facilitate both rapid learning cycles and high-quality evaluation studies for academic publication.

## Additional Files

The additional files for this article can be found as follows:

10.5334/ijic.9896.s1 Supplementary File 1. Search Strategy.

10.5334/ijic.9896.s2 Supplementary File 2. Data Extraction Instrument.

10.5334/ijic.9896.s3 Supplementary File 3. Characteristics of Included Studies.

10.5334/ijic.9896.s4 Supplementary File 4. Integrated Home and Community Care Program Description.
