# Supplementary material for: Examining the Influence of Integrated Home and Community Care Programs on Quadruple Aim and Health Equity Outcomes Across the Health Care System: A Scoping Review
Source: Int J Integr Care. 2026 Mar 19;26(1):12. doi: 10.5334/ijic.9896 (PMC13004061; doi:10.5334/ijic.9896)
Supplement: Supplementary File 1. — Search Strategy. [file ijic-26-1-9896-s1.pdf]

Supplemental File 1. Search Strategy

Administrative Burden

1	(("home and community" or "home and community based" or home based or at-home or home) adj (care or treatment or therapy or support)).tw,kf.	24959
2	exp Home Care Services, Hospital-Based/ or exp Home Health Nursing/ or exp Home Care Services/	50858
3	community health nursing/ or reablement service*.tw,kf. or integrated comprehensive care.tw,kf.	19808
4	1 or 2 or 3	77947
5	((indirect or nonclinical or nondirect) adj (activit$2 or time or care or work)).tw,kf.	424
6	(((administrative or paperwork or documentation or scheduling) adj burden) or administrative task*).tw,kf.	1259
7	5 or 6	1681
8	(4 and 7) not exp Telemedicine/ not adolescent/ not young adult/ not child/ not infant/ not pediatrics/ not (infant* or child or children or youth* or newborn* or adolescent or adolescence or pediatric*).tw,kf.	32
9	limit 8 to english language	31
10	limit 9 to yr="2013 -Current"	16

Alternate Level of Care

1	(("home and community" or "home and community based" or home based or at-home or home) adj (care or treatment or therapy or support)).tw,kf.	24959
2	exp Home Care Services, Hospital-Based/ or exp Home Health Nursing/ or exp Home Care Services/	50858
3	community health nursing/ or reablement service*.tw,kf. or integrated comprehensive care.tw,kf.	19808
4	1 or 2 or 3	77947
5	((alternat* level* adj2 care) or (delay* adj2 discharg*) or delayed transfer$1 of care or (bed$1 adj2 block$3)).tw,kf.	1796
6	(4 and 5) not exp Telemedicine/ not adolescent/ not young adult/ not child/ not infant/ not pediatrics/ not (infant* or child or children or youth* or newborn* or adolescent or adolescence or pediatric*).tw,kf.	57

7	limit 6 to (english language and yr="2013 -Current")	30

Caregiver Burden

1	(("home and community" or "home and community based" or home based or at-home or home) adj (care or treatment or therapy or support)).tw,kf.	24959
2	exp Home Care Services, Hospital-Based/ or exp Home Health Nursing/ or exp Home Care Services/	50858
3	community health nursing/ or reablement service*.tw,kf. or integrated comprehensive care.tw,kf.	19808
4	1 or 2 or 3	77947
5	((carer* or caregiving or caregiver* or care giv* or care or care person) adj1 (burden* or burnout or exhaustion or strain or stress or frustrat* or overload or fatigue)).tw,kf.	11029
6	exp Caregiver Burden/	601
7	5 or 6	11134
8	(4 and 7) not exp Telemedicine/ not adolescent/ not young adult/ not child/ not infant/ not pediatrics/ not (infant* or child or children or youth* or newborn* or adolescent or adolescence or pediatric*).tw,kf.	849
9	limit 8 to (english language and yr="2013 -Current")	332

Emergency Department Utilization

1	(("home and community" or "home and community based" or home based or at-home or home) adj (care or treatment or therapy or support)).tw,kf.	24959
2	exp Home Care Services, Hospital-Based/ or exp Home Health Nursing/ or exp Home Care Services/	50858
3	community health nursing/ or reablement service*.tw,kf. or integrated comprehensive care.tw,kf.	19808
4	1 or 2 or 3	77947
5	Emergency services, hospital/	87090
6	((emergenc* or urgent) adj (care or ward or department)).tw,kf.	126591
7	("accident and emergency" adj (ward or department)).tw,kf.	2191
8	5 or 6 or 7	162483
9	(4 and 8) not exp Telemedicine/ not adolescent/ not young adult/ not child/ not infant/ not pediatrics/ not (infant* or child or children or youth* or newborn* or	843

	adolescent or adolescence or pediatric*).tw,kf.	
10	limit 9 to (english language and yr="2013 -Current")	484

Emergency Medical Service Utilization

1	(("home and community" or "home and community based" or home based or at-home or home) adj (care or treatment or therapy or support)).tw,kf.	24959
2	exp Home Care Services, Hospital-Based/ or exp Home Health Nursing/ or exp Home Care Services/	50858
3	community health nursing/ or reablement service*.tw,kf. or integrated comprehensive care.tw,kf.	19808
4	1 or 2 or 3	77947
5	exp Paramedicine/ or emergency medical services/ or emergency medical dispatch/ or emergency medical service communication systems/ or exp Emergency medical technicians/	53277
6	((emergency medical adj (technician* or service* or practitioner*)) or (Paramedic* or EMT or EMS)).tw,kf.	64125
7	exp Ambulances/ or ambulance*.tw,kf.	18276
8	(5 or 6 or 7) not community paramedic*.tw,kf.	113457
9	(4 and 8) not exp Telemedicine/ not adolescent/ not young adult/ not child/ not infant/ not pediatrics/ not (infant* or child or children or youth* or newborn* or adolescent or adolescence or pediatric*).tw,kf.	389
10	limit 9 to english language	300
11	limit 10 to yr="2013 -Current"	102

Facility Based Long Term Care

1	((long term care or long-term care or skilled nursing or nursing or convalescent or residential care or assisted living or extended living or continuing care) adj (home or homes or facility or facilities)).tw,kf.	48111
2	exp Residential Facilities/ or exp Nursing Homes/ or exp Homes for the Aged/	58302
3	(exp Home Care Services, Hospital-Based/ or exp Home Health Nursing/ or exp Home Care Services/ or community health nursing/ or reablement service*.tw,kf. or integrated comprehensive care.tw,kf. or (("home and community" or "home and community based" or home based or at-home or home) adj (care or treatment or therapy or support)).tw,kf.) not nursing home care.tw,kf.	76155

4	1 or 2	77618
5	3 and 4	4213
6	5 not exp Telemedicine/ not adolescent/ not young adult/ not child/ not infant/ not pediatrics/ not (infant* or child or children or youth* or newborn* or adolescent or adolescence or pediatric*).tw,kf.	3806
7	limit 6 to (english language and yr="2013 -Current")	1136

Hospital Utilization

1	exp Home Care Services, Hospital-Based/ or exp Home Health Nursing/ or exp Home Care Services/	50858
2	community health nursing/ or reablement service*.tw. or integrated comprehensive care.tw,kf.	19806
3	(("home and community" or "home and community based" or home based or at-home or home) adj (care or treatment or therapy or support)).tw,kf.	24959
4	1 or 2 or 3	77945
5	exp length of stay/ or exp patient admission/ or exp patient readmission/ or hospitlization/	140579
6	((duration or length or hospital or inpatient or in-patient) adj2 stay).tw,kf.	174332
7	((hospital or patient) adj2 (admission or readmission)).tw,kf.	51290
8	5 or 6 or 7	288883
9	(4 and 8) not exp Telemedicine/ not adolescent/ not young adult/ not child/ not infant/ not pediatrics/ not (infant* or child or children or youth* or newborn* or adolescent or adolescence or pediatric*).tw,kf.	2915
10	limit 9 to english language	2591
11	limit 10 to yr="2013 -Current"	1009

Primary Care Utilization

#	Searches	Results
1	(("home and community" or "home and community based" or home based or at-home or home) adj (care or treatment or therapy or support)).tw,kf.	24959
2	exp Home Care Services, Hospital-Based/ or exp Home Health Nursing/ or exp Home Care Services/	50858

3	community health nursing/ or reablement service*.tw,kf. or integrated comprehensive care.tw,kf.	19808
4	1 or 2 or 3	77947
5	(primary adj (health care or healthcare or care)).tw,kf.	151598
6	((general or family) adj (practice* or practitioner*)).tw,kf.	102410
7	(family adj (physician* or doctor*)).tw,kf.	21364
8	Primary Health Care/ or exp General Practice/	162991
9	(5 or 6 or 7 or 8) not home-based primary care.tw,kf. not exp House Calls/	303924
10	(4 and 9) not exp Telemedicine/ not adolescent/ not young adult/ not child/ not infant/ not pediatrics/ not (infant* or child or children or youth* or newborn* or adolescent or adolescence or pediatric*).tw,kf.	3934
11	limit 10 to (english language and yr="2013 -Current")	935
