# Supplementary material for: Examining the Influence of Integrated Home and Community Care Programs on Quadruple Aim and Health Equity Outcomes Across the Health Care System: A Scoping Review
Source: Int J Integr Care. 2026 Mar 19;26(1):12. doi: 10.5334/ijic.9896 (PMC13004061; doi:10.5334/ijic.9896)
Supplement: Supplementary File 2. — Data Extraction Instrument. [file ijic-26-1-9896-s2.pdf]

Supplemental File 2. Data Extraction Instrument

Title					
Author(s)					
Year of publication					
Journal					
Aim/ research question					
Study design					
Location					
Sample size					
Participant/ population characteristics					
Services provided					
Providers of care					
Duration/ amount/ frequency of service provision					
Summary of main results					
Influence on the health system	Acute Care Service	Emergency Medical Services	Primary Care	Informal Care	Facility-Based Long-Term Care
Influence on Quadruple Aim and Health Equity	Cost	Patient and Caregiver Experience	Population Health	Provider Experience	Health Equity
