# Supplementary material for: Examining the Influence of Integrated Home and Community Care Programs on Quadruple Aim and Health Equity Outcomes Across the Health Care System: A Scoping Review
Source: Int J Integr Care. 2026 Mar 19;26(1):12. doi: 10.5334/ijic.9896 (PMC13004061; doi:10.5334/ijic.9896)
Supplement: Supplementary File 3. — Characteristics of Included Studies. [file ijic-26-1-9896-s3.pdf]

Supplemental File 3. Characteristics of Included Studies

Reference<br><br>Country	Sample Description	Study Aim and Design	Main Findings	Quadruple Aim and Health Equity Domain					Health Setting
				Value	Patient & Caregiver Experience	Population Health	Provider Experience	Health Equity	
**Comprehensive Coordinated Care**									
Compton<br>2019<br><br>Canada	Older adults (75+) ineligible for long term care with recent ED use<br>n=19	To explore the experiences of older adults and family caregivers enrolled in Home First due to a significant change in their health status.<br><br>**Qualitative**: Interviews	The intervention group perceived a reduce need to attend hospital, emergency room or primary care. Participants reported the program provided holistic care with enhanced continuity.		+				Hospital
					+				EMS
									Unpaid
					+				Primary
					+				LTCF
Maru<br>2015<br><br>Australia	Older adults hospitalized by chronic heart failure<br>n=280	To assess and compare the cost-effectiveness of home-based management for chronic heart failure with clinic-based care.<br><br>**Quantitative:** RCT	The intervention reduced the frequency of hospital stays per patient and total healthcare costs, while increasing net monetary benefit and mean quality adjusted life years.	+/x					Hospital
									EMS
									Unpaid
									Primary
									LTCF
Meunier<br>2016<br><br>USA	Older adults (55+) that were eligible for long term care<br>n=34	To examine the impact of closing of a PACE program on functional status, resource use, and patient satisfaction.<br><br>**Quantitative**: Prospective cohort	After discharge from the intervention, more ED visits, hospitalizations, and nursing home placements occurred.	+					Hospital
				+					EMS
									Unpaid
									Primary
				+					LTCF

Mougias<br>2022<br><br>Greece	Low-income older adults diagnosed with dementia and their adult primary family caregiver<br>n= 410<br>(205 patients, 205 caregivers)	To evaluate home-based intervention for people living with dementia effect on symptoms and caregiver burden/depression.<br><br>**Quantitative**: Prospective single-center longitudinal	The intervention reduced caregiver burden and depressive symptoms.						Hospital
									EMS
					+				Unpaid
									Primary
									LTCF
Penkunas<br>2018<br><br>Singapore	Caregivers (21+) of older adults enrolled in a home-based intervention<br>n= 32	To explore caregiver experiences in Aging-in-Place home-based program.<br><br>**Qualitative**: Content Analysis	The intervention reduced caregivers' secondary stressors through increased access to information and advice provided by the intervention team.						Hospital
									EMS
					+				Unpaid
									Primary
									LTCF
Vila<br>2015<br><br>Spain	Adults with one or more severe, progressive chronic condition<br>n= 293<br>(261 intervention, 32 control)	To assess if home-based program reduces costs.<br><br>**Quantitative**: Retrospective cohort	The intervention group had as reduction in number of hospital admissions, hospital LOS, and cost of care.	+					Hospital
				x					EMS
									Unpaid
									Primary
									LTCF
**Integrated Palliative Care**									
Borraccino<br>2020<br><br>Italy	Patients who received integrated home palliative care<br>n=20 611	To analyze ED visit trends in clients receiving integrated home-based palliative care.<br><br>**Quantitative**: Cross-sectional, time	The intervention led to a slight decline in emergency room visits.						Hospital
				+					EMS
									Unpaid
									Primary
									LTCF
Brännström<br>2014<br><br>Sweden	Older adults diagnosed with chronic heart failure<br>n=72<br>(36 intervention, 36 control)	To evaluate the impact of PREFER (integrated home-based palliative HF care) on symptoms, HQRL, and hospitalizations compared to usual care.	The intervention group showed significant improvements in health-related quality of life and morbidity compared to usual care, while reducing the number of hospitalization and days spent in hospitals.	+					Hospital
									EMS
									Unpaid
				+					Primary

		**Quantitative**: RCT							LTCF
Brian Cassel<br>2016<br><br>USA	Patients referred by primary care with advanced chronic illness<br>n=1443<br>(368 intervention, 1075 control)	To assess resource usage outcomes of a home-based palliative care program.<br><br>**Quantitative**: Observational	The intervention led to significantly lower frequency of hospitalization and readmission, as well as lower frequency of hospital admission in the last 30 days of life.	+					Hospital
									EMS
									Unpaid
									Primary
									LTCF
Chen<br>2015<br><br>USA	Home-bound patients who received home-based palliative care<br>n=162<br>(54 intervention, 108 control)	To evaluate hospital use, advance care planning, and mortality in clients enrolled in a home-based palliative care.<br><br>**Quantitative**: Retrospective cohort	The intervention reduced total hospital days. No differences in rates of emergency room visits.	+					Hospital
				x					EMS
									Unpaid
									Primary
									LTCF
Di Pollina<br>2017<br><br><br>Switzerland	Frail older adults (60+)<br>n=301<br>(122 intervention, 179 control)	To assess if integrated home care reduces preventable hospital/ER visits, institutionalization, and death in frail, community-dwelling older adults.<br><br>**Quantitative**: Prospective controlled trial	The intervention significantly reduces preventable hospitalization and emergency room visits allowing patients to die at home.	+/x	+				Hospital
				+					EMS
									Unpaid
									Primary
				x					LTCF
Ferroni<br>2016<br><br>Italy	Older adults (65-84) who died from non-oncological chronic diseases<br>n= 2087<br>(1071 intervention, 1016 control)	To evaluate if a home-based palliative care program affects death location and LOS in final 30 days of life.<br><br>**Quantitative**: Retrospective cohort	The intervention reduced hospital deaths and prolonged end-of-life hospitalization with two or more visits weekly.	+					Hospital
									EMS
									Unpaid
									Primary
									LTCF
Hsu<br>2021<br><br>Taiwan	Adults (18+) who had a cancer diagnosis and received home palliative care in the last six months of life<br>n=762	To compare standard care with enhanced home-based palliative care on cancer-related dyspnea.<br><br>**Quantitative**: Retrospective cohort	The intervention reduces the risk of emergency room visits related to dyspnea.						Hospital
				+					EMS
									Unpaid
									Primary

	(388 intervention, 374 control)								LTCF
Hum 2020<br><br>Singapore	Clinically stable home-dwelling older adults with more than one month predicted survival n=254	To explore the impact of home-based integrated palliative care program on caregiver burden in people living with advance dementia.<br><br>**Quantitative**: Prospective cohort	The intervention significantly improved quality of life for the intervention group and decreased caregiver burden.						Hospital
									EMS
					+				Unpaid
									Primary
									LTCF
Kentaro 2017<br><br>Japan	Home-bound patients n=106<br>(33 intervention; 72 control)	To compare end-of-life costs between physician-led home-based care program and hospital care.<br><br>**Quantitative**: Cross-sectional	There were no significant differences in average daily cost of care during the las 30 days of life between the intervention and usual care. The intervention had higher short-term costs and lower long-term costs.	+					Hospital
									EMS
									Unpaid
									Primary
									LTCF
Lustbader 2017<br><br>USA	Home-bound frail older adults with advanced heart failure n=651<br>(82 intervention, 569 control)	To evaluate cost and resource use impact of a home-based palliative care program.<br><br>**Quantitative**: Retrospective cross-sectional	The intervention reduced hospital admissions and emergency room visits during the final months of life	+					Hospital
				+					EMS
									Unpaid
									Primary
									LTCF
Mendez 2020<br><br>USA	Adults (18+) with one or more progressive chronic conditions<br>n= 22	To assess the effect of home-based palliative care program QoL, symptom control, and hospital stays.<br><br>**Quantitative**: Quasi-experimental	The intervention led to reduced average number of hospital admission days and associated with reduced ESAS symptoms	+					Hospital
									EMS
									Unpaid
									Primary
									LTCF
Mracek 2021<br><br>Canada	Deceased adults that survived longer than 180 days after diagnosis	To assess the effect of home-based palliative care on ED visits in final 30/90 days of life.	Participants enrolled in the intervention were less likely to visit the emergency room.						Hospital
				+					EMS
									Unpaid

	n= 6976	**Quantitative**: Retrospective cohort							Primary
									LTCF
Pereira 2023<br><br>Singapore	Patients of a major Singapore tertiary hospital with advanced dementia n=293 (184 intervention, 139 control)	To estimate provider costs and healthcare use in Programme Dignity (home-based palliative care for advanced dementia).<br><br>**Quantitative**: Retrospective cohort	The intervention group was less likely to visit the emergency room and be admitted to the hospital, with lower cumulative healthcare utilization costs.	+					Hospital
				+					EMS
									Unpaid
									Primary
									LTCF
Pouliot 2017<br><br>USA	Patients with a serious illness diagnosis and desired to stay at home n=123	To evaluate effectiveness of home-based palliative care program (Care Choices).<br><br>**Quantitative**: Prospective cohort	The intervention led to fewer emergency department and hospital admissions.	+					Hospital
				+					EMS
									Unpaid
									Primary
									LTCF
Punchik 2017<br><br>Israel	Home-bound patients receiving care for chronic heart failure n=196	To evaluate if home-based care for CHF reduces healthcare use and overall cost.<br><br>**Quantitative**: Retrospective cohort	The intervention led to a significantly decreases in hospitalizations, length of stays, emergency room visits and overall cost.	+					Hospital
				+					EMS
									Unpaid
									Primary
									LTCF
Quinn 2022<br><br>Canada	Community-dwelling adults (40+) with heart failure diagnosis n=245 (intervention 1172, control 1172)	To assess if coordinated home-based palliative care improves home death rates in HF patients.<br><br>**Quantitative**: Population-based matched cohort	The intervention was associated with lower risk of dying in hospital compared to usual care.	+					Hospital
				+					EMS
									Unpaid
				-					Primary
									LTCF
Ranganathan 2013<br><br>USA	Palliative eligible patients requiring skilled care needs n=16 115	To measure the impact of a home-based palliative care program on hospital readmissions.	Participants enrolled in the intervention had a lower probability of readmission to hospital.	+					Hospital
									EMS
									Unpaid

	(406 intervention, 15 709 control)	**Quantitative**: Prospective cohort							Primary
									LTCF
Rotter 2018<br><br>USA	Patients enrolled in palliative care program<br>n=35	To track symptoms and service use in a home-based palliative care program.<br><br>**Quantitative**: Quality improvement	The intervention reduced hospitalizations and readmission following discharge.	+					Hospital
									EMS
									Unpaid
									Primary
									LTCF
Sahlen 2016<br><br>Sweden	Patients diagnosed with chronic and sever heart failure n=72<br>(36 intervention, 36 control)	To evaluate cost-effectiveness of integrated HF and palliative home care program.<br><br>**Quantitative**: Randomized control trial	The intervention led to reduction in hospital admission, emergency transport, and lower total costs.	+					Hospital
				+					EMS
									Unpaid
				-					Primary
									LTCF
Scacchi 2022<br><br>Italy	Patients enrolled in the integrated home palliative care<br>n=17 983	To examine the influence of enrollment in IHPC on ED use.<br><br>**Quantitative**: Retrospective pre-/post-intervention	A statistically significantly lower rate of emergency room visits was observed after admission to intervention.						Hospital
				+					EMS
									Unpaid
									Primary
									LTCF
Skov Benthien 2018<br><br>Denmark	Adults (18+) with cancer diagnosis and their caregivers n=340<br>(intervention 162, control 160)	To investigate if home-based palliative care reduces hospital admissions for cancer patient's vs usual care.<br><br>**Quantitative**: Secondary Analysis of RCT	Caregivers in the intervention group were less likely to feel very safe caring for patients in-home.	x					Hospital
									EMS
					-				Unpaid
									Primary
									LTCF
Wang 2019<br><br>USA	Deceased older adults (65+) that were home-bound<br>n=54 751	To compare EOL care outcomes of HomePal users vs hospice-only and no-care cohorts.	The intervention was associated with fewer hospitalizations and more time at home in the final six months of life.	+					Hospital
									EMS
									Unpaid

		**Quantitative**: Prospective cohort							Primary
				+					LTCF
Yu<br>2015<br><br>Canada	Primary caregivers of terminally il cancer patients<br>n=215	To estimate EOL societal costs for hospital vs home deaths under home-based palliative care program.<br><br>**Quantitative**: Prospective cohort	No significant difference in total societal costs between the intervention and hospital death. Cost related deaths in hospitals were replaced by increased unpaid caregiver time and outpatient service costs for patients who died in their home.						Hospital
									EMS
				-					Unpaid
									Primary
									LTCF
**Preventative Care**									
Edes<br>2014<br><br>USA	Veterans with complex, chronic and disabling conditions<br>n=9524	To analyze if HBPC reduces total healthcare use/costs.<br><br>**Mixed methods:** Cost analysis & interviews	The intervention reduced hospital admission and combined hospital days, decreased net costs, improved client/caregiver relationship with the care team and had positive effects on health QoL and psychological well-being.	+	+				Hospital
									EMS
									Unpaid
									Primary
									LTCF
Edwards<br>2017<br><br>USA	Older adults (67+) that received prescriptions for diabetes<br>n=364 972	To explore HBPC's effect on avoidable hospitalizations in diabetic veterans and interactions with clinical conditions and complexities.<br><br>**Quantitative**: Retrospective cohort	The intervention was associated with fewer hospitalizations, particularly in medically complex care cases.	+/-					Hospital
									EMS
									Unpaid
									Primary
									LTCF
Gillespie<br>2021<br><br>USA	Veterans enrolled in home-based primary care<br>n=8497	To explore factors tied to avoidable hospitalizations in veterans enrolled in HBPC.<br><br>**Quantitative**: Retrospective claims-based	The intervention did not reduce hospital readmission or mortality within 6 months following hospitalization.	+					Hospital
									EMS
									Unpaid
									Primary
									LTCF
Kramer<br>2018	Rural-dwelling veterans that met the criteria for home-	To analyze rural HBPC's effect on VA access and outcomes for American Indians vs non-Indians.	The intervention led to a statistically significant decrease in the probability of hospital admissions and emergency room	+				+	Hospital
				+				+	EMS

USA	based primary care	**Quantitative**: Quasi-experimental	visits after 90 days and a year after admission to intervention was observed.						Unpaid
									Primary
									LTCF
**Restorative Care**									
Berggren 2019<br><br>Sweden	older adults (70+) discharged after hip fracture<br>n=205<br>(106 intervention, 93 control)	To examine whether home-based geriatric rehab reduces complications, readmissions, and hospital stay over 12 months compared to usual care.<br><br>**Quantitative**: Secondary analysis of RCT	No significant difference in outcome was observed.	x					Hospital
									EMS
									Unpaid
									Primary
									LTCF
Campagna 2022<br><br>Italy	Individuals enrolled in integrated home care intervention<br>n= 39 822	To study ED use patterns before, during, and after enrollment in integrated home care program.<br><br>**Quantitative**: Retrospective observational	The intervention led to short- and long-term reductions in emergency room visits.						Hospital
				+					EMS
									Unpaid
									Primary
									LTCF
Karlsson 2016<br><br>Sweden	older adults (70+) with hip fracture living at personal residence or long-term care facility<br>n=205	To examine if integrated home-based geriatric home-based rehab improves walking and reduces LOS post-hip fracture.<br><br>**Quantitative**: RCT	The intervention group had shorter length of stay in geriatric wards post-operation. - No significant differences in functionality or mortality were reported.	+					Hospital
									EMS
									Unpaid
									Primary
									LTCF
**Transitional Care**									
Ahmadi 2021<br><br>Canada	Adult (18+) thoracic surgery patients<br>n= 1288<br>(692 intervention, 596 control)	To assess impact of integrated home-based care post-thoracic surgery on LOS, readmissions, ED visits, and mortality.<br><br>**Quantitative**: Retrospective cohort	The intervention group had significantly shorter median length of stay and lower rate of emergency room visits, readmissions, and mortality.	+					Hospital
				+					EMS
									Unpaid
									Primary
									LTCF

Bellon<br>2019<br><br>USA	Older adults that meet the criteria for home care or skilled nursing facility admission<br>n=3200<br>(1900 intervention, 1300 control)	To compare 30-/90-day readmission and ED rates in older adults in a home transition program vs Medicare without home transitions.<br><br>**Quantitative**: Retrospective cohort	The intervention reduced hospital readmissions for high-risk enrollees, although 30-day observation or ED readmissions increased for medium-risk enrollees.	+					Hospital
				-					EMS
									Unpaid
									Primary
									LTCF
Chouliari<br>2014<br><br>England	Healthcare professionals, managerial staff, and individuals that made referrals to the intervention<br>n=35	To explore provider experiences on stroke early supported discharge implementation and impact.<br><br>**Qualitative**: Interviews	Most participants perceived benefits of the intervention.	+			+/-		Hospital
									EMS
									Unpaid
									Primary
									LTCF
Cousse<br>2019<br><br>France	Individuals diagnosed with COPD<br>n=264<br>(62 intervention, 202 control)	To test if PRADO-BPCO home care program reduces 28-day COPD readmissions.<br><br>**Quantitative**: Retrospective cohort	Inpatient stay was shorter in intervention group compared to control. Readmission or death rate at 28 days was similar between groups.	+/x					Hospital
									EMS
									Unpaid
									Primary
									LTCF
Deng<br>2020<br><br>China	Rural-dwelling stroke survivors<br>n=98<br>(49 intervention, 49 control)	To assess 8-week integrated transitional care program's effect on stroke survivor outcomes.<br><br>**Quantitative**: RCT	The intervention group showed significant improvement in the burden of illness.						Hospital
									EMS
					+				Unpaid
									Primary
									LTCF
Guertin 2017<br><br>Canada	Community-dwelling patients with a hospital admission driven by COPD n=344<br>(76 intervention, 268 control)	To assess the integrated comprehensive care program bundle's effect on hospital utilization outcomes in COPD patients.<br><br>**Quantitative**: Retrospective observational cohort	No statistically significant difference in readmission was observed.	+/x					Hospital
									EMS
									Unpaid
									Primary
									LTCF

Jepma<br>2021<br><br>Netherlands	Community-dwelling cardiac patients<br>n= 306<br>(153 intervention, 153 control)	To test impact of home-based cardiac care transitions program on readmissions and mortality compared to usual care.<br><br>**Quantitative**: RCT	The intervention did not reduce hospital readmission or mortality within 6 months following hospitalization.	x					Hospital
									EMS
									Unpaid
									Primary
									LTCF
Low<br>2015<br><br>Singapore	Adults with 3+ chronic medical conditions and limited mobility<br>n=262	To assess impact of transitional home care program on hospital use.<br><br>**Quantitative**: Quasi-experimental	Significant reduction in hospital admission, hospital length of stay, and emergency room visits was observed.	+					Hospital
				+					EMS
									Unpaid
									Primary
									LTCF
Low<br>2017<br><br>Singapore	Patients with functional dependence admitted to hospital n=1 166<br>(541 intervention, 625 control)	To test if IPU-based transitional home care lowers 30-/90-day readmissions in functionally dependent patients.<br><br>**Quantitative**: Retrospective cohort	A statistically significant reduction in 30-day readmissions and emergency department visits in patients admitted to intervention was observed.	+					Hospital
				+					EMS
									Unpaid
									Primary
									LTCF
Markle Reid<br>2020<br><br>Canada	Older adults (55+) with confirmed stroke within the past 12 months<br>n=30	To test feasibility and effects of integrated transitional stroke care program in multimorbid older adults compared to outpatient care.<br><br>**Mixed methods:** Prospective one-group pretest/post-test & focus groups/engagement sessions	There was a significant reduction in the total per person use and costs of health services in the intervention group.	+					Hospital
				+					EMS
									Unpaid
									Primary
									LTCF
Simpson<br>2019<br><br>USA	Older adults (65+) admitted to homecare<br>n=766<br>(194 intervention, 617 control)	To assess the hospital readmission risk with or without bundled program targeting older adults post-discharge.<br><br>**Quantitative**: Matched case-control	The intervention was associated with fewer hospitalizations and skilled nursing facility stays and more time at home in the final six months of life.	+					Hospital
				x					EMS
									Unpaid
									Primary
				x					LTCF

**Legend**					
+	Positive influence	-	Negative influence	x	No difference
+/x	Mixed positive/no influence	-/x	Mixed negative/no influence	+/-	Mixed influence
