# Supplementary material for: Examining the Influence of Integrated Home and Community Care Programs on Quadruple Aim and Health Equity Outcomes Across the Health Care System: A Scoping Review
Source: Int J Integr Care. 2026 Mar 19;26(1):12. doi: 10.5334/ijic.9896 (PMC13004061; doi:10.5334/ijic.9896)
Supplement: Supplementary File 4. — Integrated Home and Community Care Program Description. [file ijic-26-1-9896-s4.pdf]

Supplemental 4. Integrated Home and Community Care Program Description

Study	Program Activities	Program Team													Program Type
		Coordinator/ Case Manager	Nurse	Primary Care Provider	Specialist Physician	OT	PT	Dietician	Social Worker	Unregulated Provider	SLP	Mental Health Provider	Pharmacist	Other	
Ahmadi 2021	Case Management/Coordination<br>Comprehensive Assessment<br>Care Planning<br>Referral and System Navigation<br>Health Education<br>Medication Optimization and Management<br>Home Health Nursing<br>Therapies<br>24/7 Call Line	X	X			X	X	X	X	X	X				Transitional
Bellon 2019	Case Management/Coordination<br>Care Planning<br>Advance Care Planning<br>Referral and System Navigation<br>Health Education<br>Medication Optimization and Management<br>Support for IADL/ADLs<br>Home Health Nursing	X		X	X				X				X	X	Transitional

Berggren 2019	Case Management/Coordination<br>Comprehensive Assessment<br>Care Planning<br>Medication Optimization and Management<br>Therapies<br>Environment Modifications		X		X	X	X	X	X					X	Restorative
Borraccino 2020	Care Planning<br>Home Health Nursing<br>Psychosocial Services<br>Therapies		X	X	X				X					X	Palliative
Bronnstrom 2014	Case Management/Coordination<br>Comprehensive Assessment<br>Care Planning<br>Advance Care Planning<br>Referral and System Navigation<br>Health Education<br>Home Health Nursing		X		X	X	X								Palliative
Campagna 2022	Home Health Nursing<br>Psychosocial Services<br>Environment Modification		X	X	X				X					X	Restorative
Brian Cassel 2016	Comprehensive Assessment<br>Advance Care Planning<br>Health Education<br>Medication Optimization and Management<br>Support for IADL/ADLs<br>Home Health Nursing<br>Psychosocial Services<br>24/7 Call Line		X		X				X					X	Palliative

Chen<br>2015	Case Management/Coordination<br>Care Planning<br>Advance Care Planning<br>Health Education<br>Medication Optimization and Management<br>Home Health Nursing<br>Psychosocial Services<br>Environment Modifications	X	X		X				X				X	X	Palliative
Chouliara<br>2014	Not specified		X		X	X	X		X	X	X	X		X	Transitional
Compton<br>2019	Case Management/Coordination<br>Health Education<br>Support for IADL/ADLs<br>Home Health Nursing<br>Psychosocial Services		X			X	X	X	X	X	X	X	X	X	Comprehensive Coordinated Care
Cousse<br>2019	Health Education<br>Psychosocial Services<br>Therapies	X	X	X	X		X								Transitional
Deng<br>2020	Health Education<br>Psychosocial Services<br>Therapies	X	X	X	X									X	Transitional
DiPollina<br>2017	Comprehensive Assessment<br>Medication Optimization and Management<br>Support for IADL/ADLs<br>Home Health Nursing<br>24/7 Call Line		X	X	X	X	X	X	X			X		X	Palliative

Edes 2014	Health Education<br>Medication Optimization and Management<br>Home Health Nursing		X	X		X	X	X							Preventative
Edwards 2017	VA HBPC (not specified)														Preventative
Ferroni 2016	not specified		X	X	X										Palliative
Gillespie 2021	Not specified														Preventative
Guertin 2017	Not specified	Not specified													Transitional
Hsu 2021	Comprehensive Assessment<br>Health Education<br>Medication Optimization and Management<br>Home Health Nursing<br>24/7 Call Line		X	X					X					X	Palliative
Hum 2020	Comprehensive Assessment<br>Health Education<br>Medication Optimization and Management<br>Psychosocial Services<br>24/7 Call Line		X		X				X						Palliative

Jepma 2021	Case Management/Coordination<br>Comprehensive Assessment<br>Care Planning<br>Health Education<br>Medication Optimization and Management<br>Therapies		X	X	X		X						X		Transitional
Karlsson 2016	Care Planning<br>Medication Optimization and Management<br>Support for IADL/ADLs<br>Home Health Nursing<br>Therapies<br>Environment Modifications		X		X	X	X							X	Restorative
Kentaro Kinjo 2017	Health Education<br>Medication Optimization and Management<br>Support for IADL/ADLs<br>Home Health Nursing<br>Therapies		X	X		X	X			X				X	Palliative
Kramer 2018	Not specified	Not specified													Preventative
Low 2017	Case Management/Coordination<br>Comprehensive Assessment<br>Health Education<br>Medication Optimization and Management<br>Home Health Nursing<br>Psychosocial Services<br>Environment Modification	X		X		X	X		X		X				Transitional

Low<br>2015	Comprehensive Assessment<br>Care Planning<br>Health Education<br>Medication Optimization and Management<br>Home Health Nursing	X	X	X		X	X		X		X		X	X	Transitional
Lustbader<br>2017	Advance Care Planning<br>Home Health Nursing<br>Psychosocial Services<br>24/7 Call Line		X	X					X	X				X	Palliative
Markle-Reid<br>2020	Case Management/Coordination<br>Comprehensive Assessment<br>Health Education<br>Medication Optimization and Management<br>Psychosocial Services	X	X			X	X		X		X				Transitional
Maru<br>2015	Case Management/Coordination<br>Comprehensive Assessment<br>Care Planning<br>Health Education		X	X	X									X	Comprehensive Coordinated Care
Mendez<br>2020	Not specified	X	X	X	X		X		X	X				X	Palliative
Meunier<br>2016	Home Health Nursing	X	X	X		X	X	X	X					X	Comprehensive Coordinated Care

Mougias<br>2022	Comprehensive Assessment<br>Care Planning<br>Health Education<br>Medication Optimization and Management<br>Psychosocial Services<br>Therapies	X				X			X	X		X			Comprehensive Coordinated Care
Mracek<br>2021	Support for IADL/ADLs<br>Home Health Nursing<br>Psychosocial Services<br>24/7 Call Line	X		X										X	Palliative
Penkunas<br>2018	Comprehensive Assessment<br>Home Health Nursing		X		X				X	X		X			Comprehensive Coordinated Care
Pereira<br>2023	Case Management/Coordination<br>Comprehensive Assessment<br>Home Health Nursing<br>Psychosocial Services<br>24/7 Call Line		X	X					X						Palliative
Pouliot<br>2017	Advance Care Planning<br>Health Education<br>Medication Optimization and Management<br>Home Health Nursing<br>Psychosocial Services		X						X					X	Palliative

Punchik 2017	Comprehensive Assessment<br>Care Planning<br>Support for IADL/ADLs<br>Home Health Nursing<br>Psychosocial Services<br>Therapies<br>Environment Modifications<br>24/7 Call Line		X	X	X	X	X		X					X	Palliative
Quinn 2022	Case Management/Coordination<br>Comprehensive Assessment<br>Medication Optimization and Management<br>Support for IADL/ADLs<br>Home Health Nursing<br>Environment Modifications	X	X	X	X									X	Palliative
Ranganathan 2013	Home Health Nursing<br>24/7 Call Line		X	X					X					X	Palliative
Rotter 2018	Comprehensive Assessment<br>Advance Care Planning<br>Medication Optimization and Management<br>Home Health Nursing<br>Psychosocial Services		X	X					X					X	Palliative

Sahlen 2016	Case Management/Coordination<br>Advance Care Planning<br>Health Education<br>Support for IADL/ADLs<br>Home Health Nursing<br>Psychosocial Services		X		X	X	X								Palliative
Scacchi 2022	Care Planning<br>Home Health Nursing<br>Psychosocial Services<br>Therapies<br>Environment Modifications	Not specified													Palliative
Simpson 2019	Support for IADL/ADLs<br>Therapies	X	X	X	X	X	X		X	X				X	Transitional
SkovBenthien 2018	Comprehensive Assessment<br>Advance Care Planning<br>Home Health Nursing<br>Psychosocial Services			X	X							X		X	Palliative
Vila 2015	Case Management/Coordination<br>Comprehensive Assessment<br>Care Planning<br>Health Education<br>Medication Optimization and Management<br>Support for IADL/ADLs<br>Home Health Nursing<br>Psychosocial Services<br>Therapies	X	X	X	X										Comprehensive Coordinated Care

Wang<br>2019	Comprehensive Assessment<br>Health Education<br>Support for IADL/ADLs<br>Home Health Nursing<br>Psychosocial Services<br>Therapies	X	X		X				X					X	Palliative
Yu<br>2015	Support for IADL/ADLs<br>Home Health Nursing		X		X					X					Palliative
